# DNA demethylation in the hypothalamus promotes transcription of *Agtr1a* and *Slc12a2* and hypertension development

**DOI:** 10.1016/j.jbc.2023.105597

**Published:** 2023-12-29

**Authors:** Krishna Ghosh, Jing-Jing Zhou, Jian-Ying Shao, Shao-Rui Chen, Hui-Lin Pan

**Affiliations:** Center for Neuroscience and Pain Research, Department of Anesthesiology and Perioperative Medicine, The University of Texas MD Anderson Cancer Center, Houston, Texas, USA

**Keywords:** chromatin plasticity, DNA methyltransferase, epigenetics, sympathetic nervous system, ten-eleven translocation methylcytosine dioxygenase, neurogenic hypertension

## Abstract

Increased expression of angiotensin II AT_1A_ receptor (encoded by *Agtr1a*) and Na^+^-K^+^-Cl^−^ cotransporter-1 (NKCC1, encoded by *Slc12a2*) in the hypothalamic paraventricular nucleus (PVN) contributes to hypertension development. However, little is known about their transcriptional control in the PVN in hypertension. DNA methylation is a critical epigenetic mechanism that regulates gene expression. Here, we determined whether transcriptional activation of *Agtr1a* and *Slc12a2* results from altered DNA methylation in spontaneously hypertensive rats (SHR). Methylated DNA immunoprecipitation and bisulfite sequencing-PCR showed that CpG methylation at *Agtr1a* and *Slc12a2* promoters in the PVN was progressively diminished in SHR compared with normotensive Wistar-Kyoto rats (WKY). Chromatin immunoprecipitation-quantitative PCR revealed that enrichment of DNA methyltransferases (DNMT1 and DNMT3A) and methyl-CpG binding protein 2, a DNA methylation reader protein, at *Agtr1a* and *Slc12a2* promoters in the PVN was profoundly reduced in SHR compared with WKY. By contrast, the abundance of ten-eleven translocation enzymes (TET1-3) at *Agtr1a* and *Slc12a2* promoters in the PVN was much greater in SHR than in WKY. Furthermore, microinjecting of RG108, a selective DNMT inhibitor, into the PVN of WKY increased arterial blood pressure and correspondingly potentiated *Agtr1a* and *Slc12a2* mRNA levels in the PVN. Conversely, microinjection of C35, a specific TET inhibitor, into the PVN of SHR markedly reduced arterial blood pressure, accompanied by a decrease in *Agtr1a* and *Slc12a2* mRNA levels in the PVN. Collectively, our findings suggest that DNA hypomethylation resulting from the DNMT/TET switch at gene promoters in the PVN promotes transcription of *Agtr1a* and *Slc12a2* and hypertension development.

Hypertension is a highly prevalent, insidious disorder and a major risk factor for diseases such as ischemic heart disease, stroke, and kidney failure. Primary hypertension, or hypertension of unknown cause, arises from complex gene-environment interactions and accounts for 90 to 95% of adult cases. The development of hypertension is closely linked to increased vasoconstrictive sympathetic outflow emanating from the brain (1, 2, 3). Within the brain, the paraventricular nucleus (PVN) of the hypothalamus, through its projections to sympathetically related neurons in the brainstem and spinal cord, serves as a crucial site for generating increased sympathetic drive in hypertension (3, 4). Spontaneously hypertensive rats (SHR), a commonly used model of primary hypertension, exhibit an age-dependent increase in arterial blood pressure (ABP) starting around 5 to 6 weeks of age, reaching a stable hypertensive state at 13 to 15 weeks (5). Electrolytic lesion of the PVN in young SHR blunts hypertension development (6, 7), and pharmacological inhibition of the PVN reduces ABP and sympathetic nerve discharges in adult SHR (8, 9, 10). Furthermore, transplantation of embryonic hypothalamic tissue containing the PVN from SHR to normotensive rats leads to hypertension in the recipient rats (11). Despite this knowledge, the molecular mechanisms engaged in the development of age-dependent neurogenic hypertension remain enigmatic.

Epigenetic modifications involve the introduction or removal of specific chemical moieties on chromatin, resulting in either permissive or repressive transcriptional states that regulate gene expression. DNA methylation, a reversible process regulated by DNA methyltransferases (DNMTs), entails the addition of 5-methylcytosine (5mC) marks on CpG dinucleotides. Conversely, DNA demethylation, crucial for gene transcription, is facilitated by ten-eleven translocation (TET) methylcytosine dioxygenases, which remove the 5mC marks (12, 13). In addition, methyl-CpG binding protein 2 (MeCP2) plays a role in reading the DNA methylation marks and facilitates interactions between various cofactors and transcription factors within chromatin, thereby influencing DNA methylation-mediated gene transcription (14). Reduced DNA methylation at gene promoters generally promotes transcriptional activation, whereas increased methylation of the CpGs often silences gene transcription. However, our knowledge regarding the epigenetic reprogramming in the brain contributing to hypertension development is very limited.

An imbalance between excitatory glutaminergic input and inhibitory GABAergic input within the PVN is pivotal in the increased sympathetic vasomotor activity in SHR (8, 15, 16, 17, 18). Upregulation of AT_1A_ receptors, encoded by the *Agtr1a* gene, in the PVN contributes critically to hypertension development. In this regard, AT_1A_ receptor stimulation by angiotensin II enhances excitatory glutaminergic input to PVN presympathetic neurons, resulting in augmented sympathetic nerve discharges (19, 20). Additionally, Na^+^-K^+^-Cl^−^ cotransporter-1 (NKCC1), encoded by the *Slc12a2* gene, critically regulates intercellular chloride levels and maintains normal GABAergic synaptic inhibition (21). In SHR, NKCC1 upregulation in the PVN disrupts chloride homeostasis, diminishing GABAergic inhibition of PVN presympathetic neurons and resulting in increased sympathetic outflow (22). At present, the epigenetic mechanisms responsible for the upregulation of AT_1A_ receptors and NKCC1 in the PVN during hypertension development have not been identified.

To address these gaps in knowledge, we determined whether DNA methylation plays a role in the transcriptional activation of *Agtr1a* and *Slc12a2* in the PVN of SHR. Our study reveals for the first time that the increased transcription of *Agtr1a* and *Slc12a2* is associated with their promoter DNA hypomethylation, resulting from coordinated changes in the enrichment of DNMTs, MeCP2, and TETs at their promoters in the PVN in hypertension. Importantly, DNMTs and TETs within the PVN actively control ABP and transcription of *Agtr1a* and *Slc12a2*. These findings provide novel insight into the brain epigenetic reprogramming involved in hypertension development.

## Results

### Age-dependent increases in ABP and transcripts of *Agtr1a* and *Slc12a2* in the PVN of SHR

**Figure 1:**
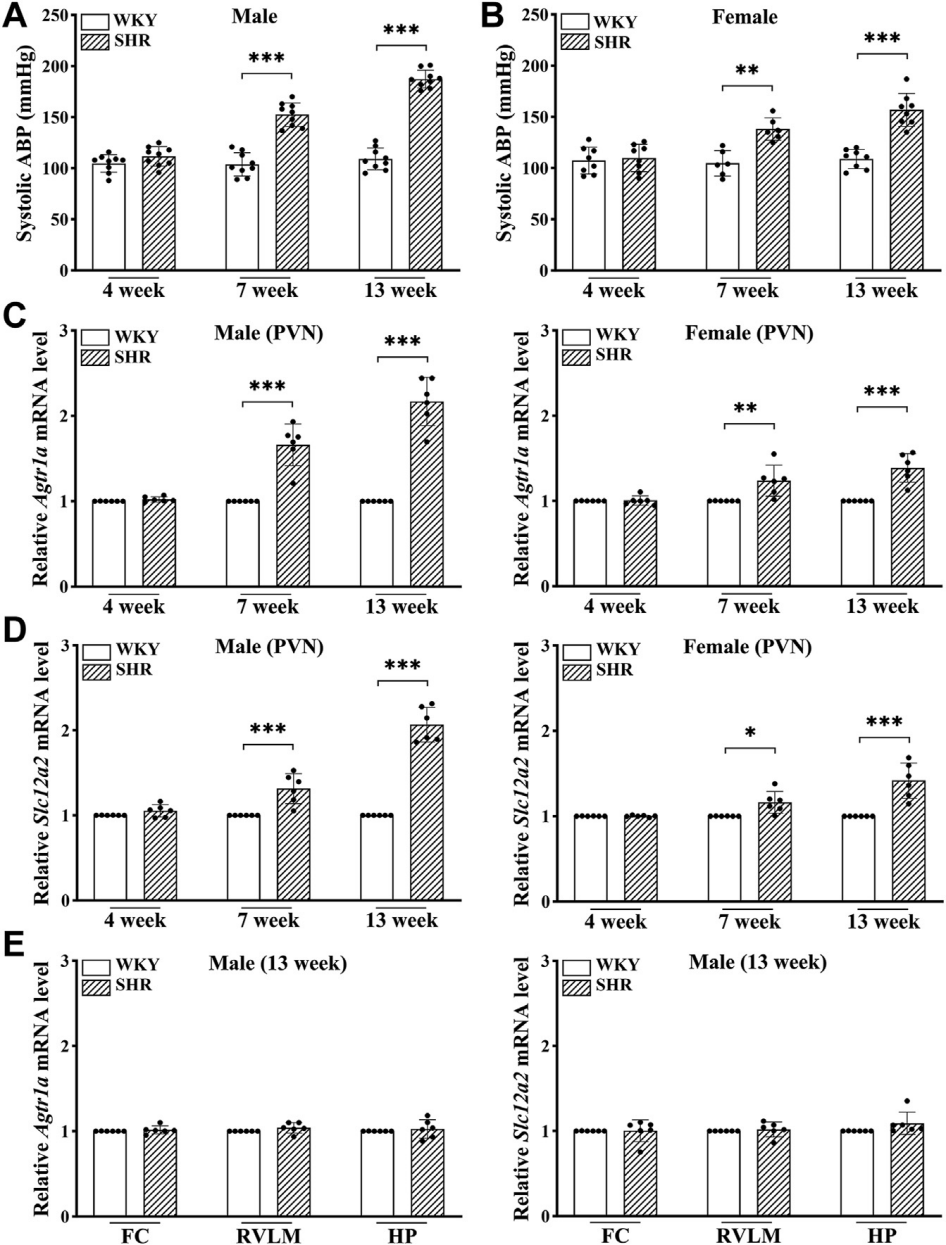
**Age-dependent increases in arterial blood pressure and transcription of *Agtr1a* and *Slc12a2* in the PVN of SHR.***A* and *B*, systolic arterial blood pressure (ABP) of 4-, 7-, and 13-week-old male (*A*) and female (*B*) WKY and SHR (n = 9 rats per group) measured using the noninvasive tail-cuff system. *C* and *D*, relative mRNA levels of *Agtr1a* (*C*) and *Slc12a2* (*D*) in the PVN of 4-, 7-, and 13-week-old male and female WKY and SHR (n = 6 rats per group). *E*, relative mRNA levels of *Agtr1a* and *Slc12a2* in the frontal cortex (FC), rostral ventrolateral medulla (RVLM), and hippocampus (HP) of 13-week-old male WKY and SHR (n = 6 rats per group). Data are presented as mean ± SD. ∗*p* < 0.05, ∗∗*p* < 0.01, ∗∗∗*p* < 0.001 (two-way ANOVA followed by Bonferroni's *post hoc* test in *A* and *B*; two-tailed Student’s *t* test in *C*–*E*). PVN, paraventricular nucleus; SHR, spontaneously hypertensive rats; WKY, Wistar-Kyoto rats.

Measurement of systolic ABP with a tail-cuff device showed that systolic ABP was similar in male Wistar-Kyoto rats (WKY) and SHR at 4 weeks of age. However, at 7 weeks old, systolic ABP was significantly elevated in male SHR compared to male WKY (n = 9 rats per group, F(2,32) = 57.04, *p* < 0.001; Figure 1*A*). By 13 weeks, the difference became even more pronounced, with systolic ABP much higher in SHR compared to WKY (n = 9 rats per group; F(2,32) = 57.04, *p* < 0.001; Fig. 1*A*). Similarly, female SHR showed an age-dependent increase in systolic ABP at 7 and 13 weeks of age compared to age-matched female WKY (n = 6 rats in 7-week old female WKY and SHR, n = 8 rats in all other groups; Fig. 1*B*).

To determine age-dependent changes in the expression level of AT_1A_ receptors and NKCC1 in the PVN in male and female WKY and SHR, we used quantitative PCR (qPCR) to measure the mRNA level of *Agtr1a* and *Slc12a2*. The mRNA levels of *Agtr1a* and *Slc12a2* in the PVN did not differ significantly between 4-week-old WKY and SHR of both sexes. The mRNA level of *Agtr1a* in the PVN was significantly greater in male SHR at 7 weeks old (t (10) = 6.596, *p* < 0.001) and 13 weeks old (t (10) = 10.12, *p* < 0.001) compared to age-matched WKY (n = 6 rats per group, Fig. 1*C*). Additionally, the *Slc12a2* mRNA level in the PVN was significantly elevated in male SHR at 7 (t (10) = 4.376, *p* < 0.001) and 13 (t (10) = 12.81, *p* < 0.001) weeks of age compared to age-matched male WKY (n = 6 rats per group, Fig. 1*D*).

In female SHR, the mRNA level of *Agtr1a* (7-week-old: t (10) = 3.214, *p* = 0.0093; 13-week-old: t (10) = 5.662, *p* < 0.001) and *Slc12a2* (7-week-old: t (10) = 3.147, *p* = 0.0104; 13-week-old: t (10) = 4.955, *p* < 0.001) in the PVN was also significantly increased at 7 and 13 weeks of age compared to age-matched female WKY (n = 6 rats per group; Fig. 1, *C* and *D*). At 7 weeks of age, the rise in ABP and mRNA levels of *Agtr1a* and *Slc12a2* was less pronounced in female SHR compared to male SHR (Fig. 1, *A*–*D*). Because both male and female SHR exhibit similar age-dependent increases in ABP and *Agtr1a* and *Slc12a2* transcripts in the PVN, we mainly used 13-week-old male SHR and WKY for the rest of the study.

To determine whether increased *Agtr1a* and *Slc12a2* transcripts occur in other brain regions in adult SHR, we measured mRNA levels of *Agtr1a* and *Slc12a2* in the frontal cortex, rostral ventrolateral medulla, and hippocampus, which showed no significant differences between 13-week-old male SHR and WKY (n = 6 rats per group; Fig. 1*E*). These results suggest that the age-dependent elevation in ABP in SHR is associated with progressive increases in the transcription of *Agtr1a* and *Slc12a2* predominantly in the PVN.

### DNA methylation at the *Agtr1a* promoter in the PVN is diminished in SHR

**Figure 2:**
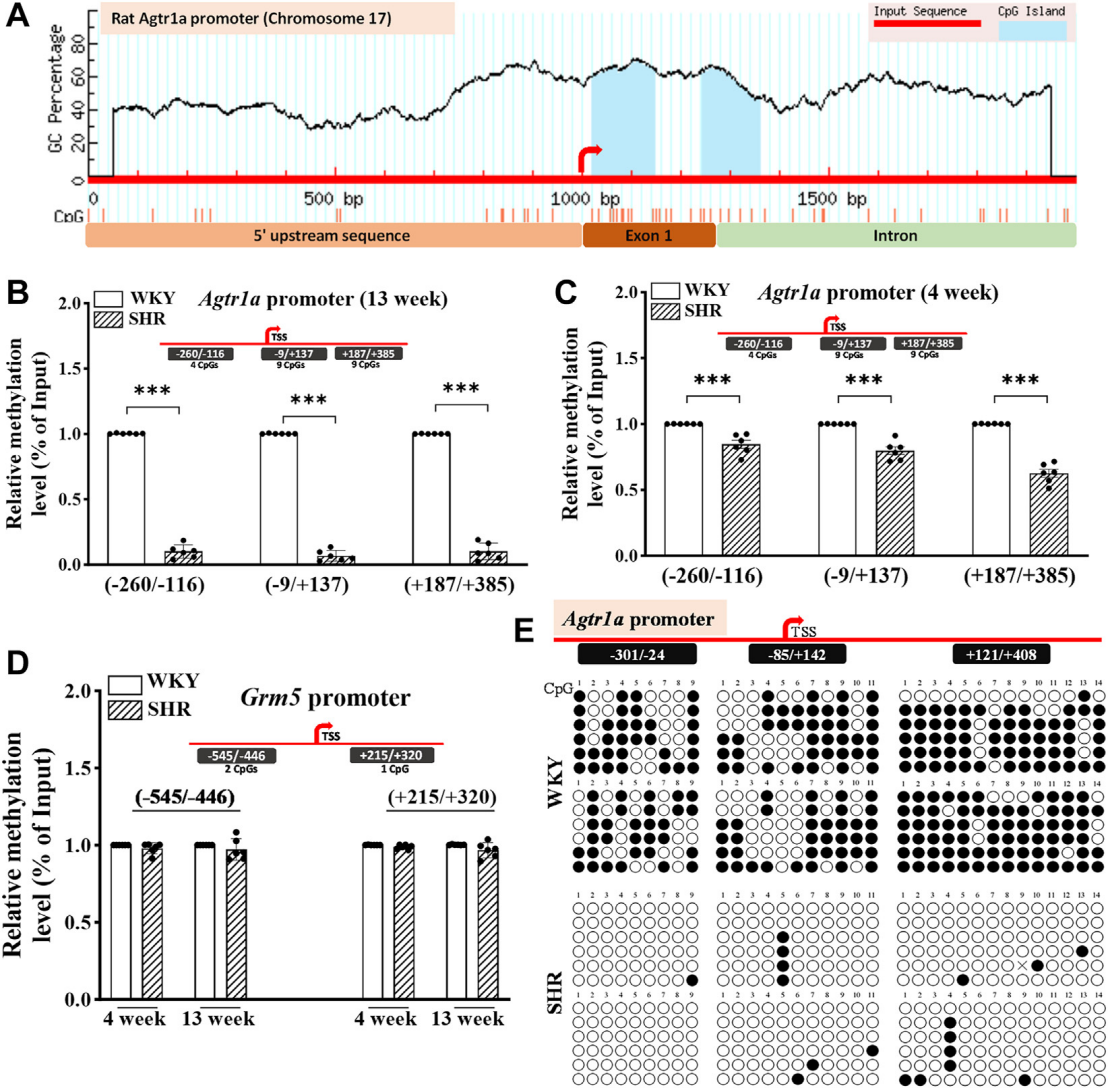
**CpG island prediction and diminished DNA methylation at the *Agtr1a* promoter in the PVN of SHR.***A*, the 1000 bp upstream/downstream DNA sequence from the first exon of the rat *Agtr1a* gene on chromosome 17 was retrieved from NCBI gene database and used to predict CpG island(s) using MethPrimer program. *B* and *C*, DNA methylation levels, measured using MeDIP-qPCR, at the indicated loci of *Agtr1a* promoter in the PVN of 13-week-old (*B*) and 4-week-old (*C*) male WKY and SHR (n = 6 rats per group). *D*, DNA methylation levels, measured by MeDIP-qPCR, at the indicated loci of *Grm5* promoter in the PVN of 4-week-old and 13-week-old male WKY and SHR (n = 6 rats per group). Data in (*B*–*D*) are represented as mean ± SD. ∗∗∗*p* < 0.001 (two-tailed Student’s *t* test). *E*, methylation status of the CpG dinucleotides, measured using bisulfite sequencing-PCR assays, in the indicated region at the *Agtr1a* promoter in the PVN of 13-week-old WKY and SHR. Each region in the top schematic diagram indicates genomic coordinates relative to the transcription start site (TSS). The *filled* and *empty circles* represent methylated and unmethylated CpGs, respectively. The bisulfite sequencing assay was repeated independently twice with sequencing of at least six clones each time. Grm5, metabotropic glutamate receptor 5; MeDIP, methylated DNA immunoprecipitation; PVN, paraventricular nucleus; qPCR, quantitative PCR; SHR, spontaneously hypertensive rats; WKY, Wistar-Kyoto rats.

DNA methylation is a common epigenetic modification that often occurs at CpG islands surrounding gene promoters. CpG islands are short DNA stretches with a high density of CpG dinucleotides, and the cytosine residues in these regions can be methylated or unmethylated in higher-order eukaryotic genomes, playing a key role in gene transcription regulation (23). In neural tissues, DNA hypomethylation is associated with increased gene expression (24, 25). We thus determined whether DNA methylation at the promoters of *Agtr1a* and *Slc12a2* in the PVN is altered in SHR. Bioinformatic analysis revealed the presence of two CpG islands close to the transcription start site (TSS) of the *Agtr1a* gene on rat chromosome 17 (Fig. 2*A*). One CpG island spans the region downstream from the TSS (+23 to +149 bp) covering the first exon of 129 bp, whereas the other CpG island is located within the first intron (+245 to +362 bp). Additionally, a few CpGs were found in the 5′-upstream DNA sequence relative to the TSS.

To determine the DNA methylation level in the *Agtr1a* promoter region in the PVN, we used methylated DNA immunoprecipitation (MeDIP) followed by qPCR analysis. MeDIP-qPCR allows us to quantify the relative enrichment of methylated DNA using the chromatin immunoprecipitated with an anti-5mC antibody. The analysis revealed a profound reduction in DNA methylation at the *Agtr1a* promoter in the PVN of 13-week-old SHR compared to age-matched WKY. Specifically, the relative enrichment of 5mC in three regions of the promoter was largely reduced in SHR compared to WKY (Fig. 2*B*). Also, the relative enrichment of 5mC in the upstream region from −260 to −116 bp, which includes four CpGs in the PVN, was markedly reduced in SHR compared to WKY (t (10) = 43.14, *p* < 0.001; n = 6 rats per group; Fig. 2*B*). Moreover, the enrichment of methylated CpGs in the regions from −9 to +137 bp (containing nine CpGs; t (10) = 55.97, *p* < 0.001; n = 6 rats per group) and from +187 to +385 bp (containing nine CpGs; t (10) = 34.39, *p* < 0.001; n = 6 rats per group) of the *Agtr1a* promoter in the PVN was diminished in SHR compared to WKY (Fig. 2*B*).

To determine whether DNA demethylation at the *Agtr1a* promoter in the PVN occurs prior to the onset of hypertension, we measured DNA methylation at the *Agtr1a* promoter in the PVN in 4-week-old “normotensive” SHR. MeDIP-qPCR assay showed that DNA methylation levels in the same three regions of the *Agtr1a* promoter in the PVN exhibited a small, yet statistically significant reduction in a 4-week-old male SHR compared to an age-matched male WKY (n = 6 rats per group; Fig. 2*C*). By contrast, MeDIP-qPCR analysis of two distinct regions (−545 to −446 bp and +215 to +320 bp) of the promoter of metabotropic glutamate receptor 5 (*Grm5*) showed no significant difference in the DNA methylation level in the PVN between SHR and WKY at 4 or 13 weeks of age (n = 6 rats per group; Fig. 2*D*).

To identify the altered methylation levels at the single base pair resolution, we conducted bisulfite sequencing-PCR (BSP) using the genomic DNA isolated from the PVN of 13-week-old SHR and WKY. The DNA was treated with sodium bisulfite that converts unmethylated cytosines into uracil, leaving the methylated cytosines intact (26). Specific primers were used to amplify a region of interest within the *Agtr1a* promoter *via* PCR, and the amplified DNA was subcloned into a plasmid vector. By subjecting these clones to Sanger sequencing, we could identify the methylated and unmethylated alleles from the PVN. Thus, the BSP assay allows us to distinguish methylated cytosines from unmethylated ones within the target region. The BSP analysis revealed a significant reduction in DNA methylation at nine CpGs within the −301 to −24 bp region, 11 CpGs within the −85 to +142 bp region, and 14 CpGs within the +121 to +408 bp region of the *Agtr1a* promoter in the PVN of SHR compared to WKY (Fig. 2*E*). These findings indicate that CpGs in the vicinity of the TSS of *Agtr1a* in the PVN become largely unmethylated in SHR, creating a “permissive” chromatin state that enhances *Agtr1a* transcription.

### CpGs at the *Slc12a2* promoter in the PVN become unmethylated in SHR

**Figure 3:**
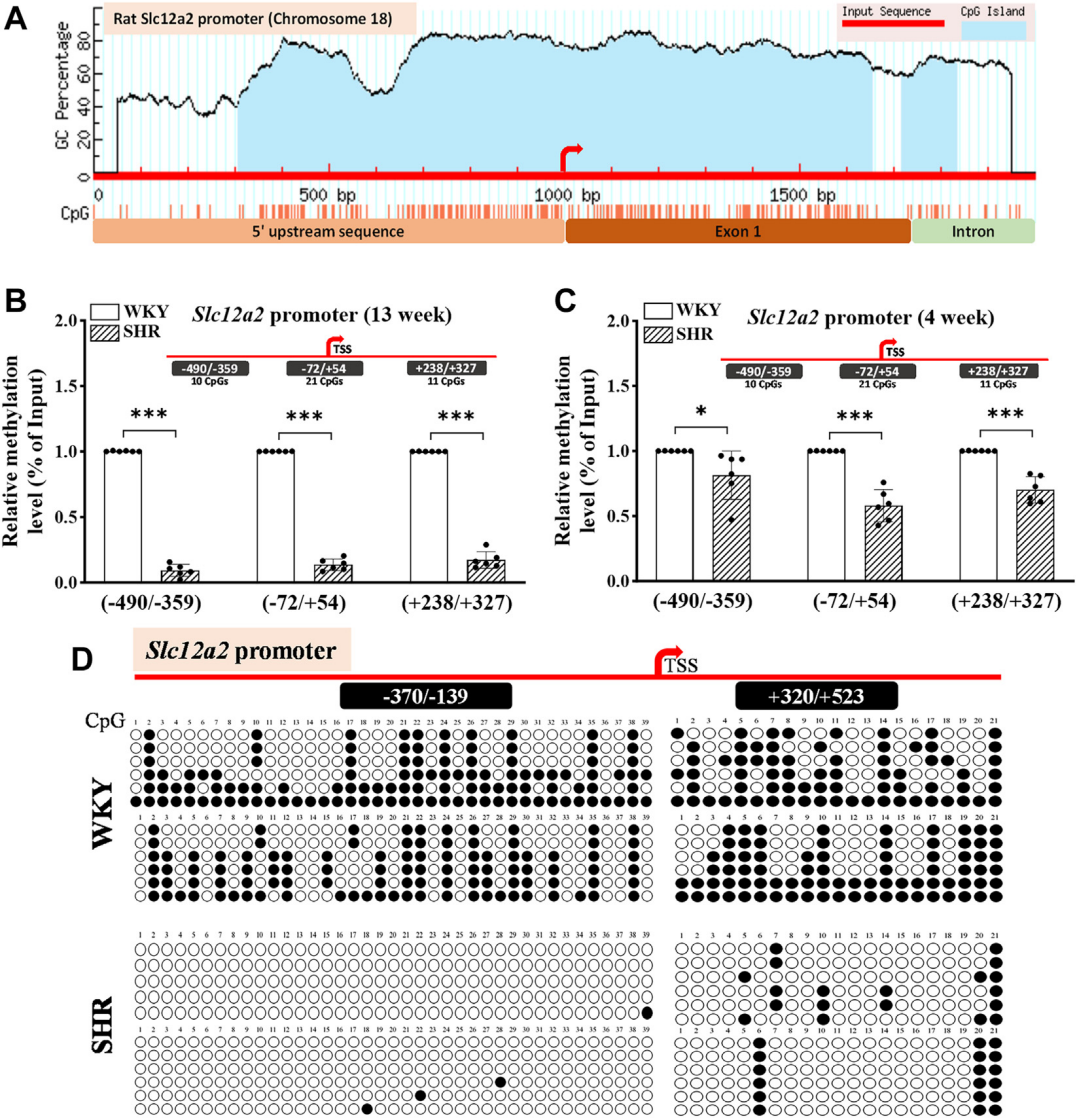
**CpG island prediction and DNA hypomethylation at the *Slc12a2* promoter in the PVN of SHR.***A*, the 2000 bp upstream/downstream DNA sequence from the first exon of the rat *Slc12a2* gene on chromosome 18 was retrieved from NCBI gene database and used to predict CpG island(s) using MethPrimer program. *B* and *C*, DNA methylation levels, quantified using MeDIP-qPCR, at the indicated loci of *Slc12a2* gene promoter in the PVN of 13-week-old (*B*) and 4-week-old (*C*) male WKY and SHR (n = 6 rats per group). Data in (*A*–*C*) are represented as mean ± SD. ∗*p* < 0.05, ∗∗∗*p* < 0.001 (two-tailed Student’s *t* test). *D*, methylation status of the CpG dinucleotides, measured using bisulfite sequencing-PCR assays, in the indicated region at the *Slc12a2* promoter in the PVN of 13-week-old WKY and SHR. Each region in the top schematic diagram indicates genomic coordinates relative to the transcription start site (TSS). The *filled* and *empty circles* represent methylated and unmethylated CpGs, respectively. The bisulfite sequencing assay was repeated independently twice with sequencing of at least six clones each time. MeDIP, methylated DNA immunoprecipitation; PVN, paraventricular nucleus; qPCR, quantitative PCR; SHR, spontaneously hypertensive rats; WKY, Wistar-Kyoto rats.

Subsequently, we examined the 2000-base pair region surrounding the TSS of the rat *Slc12a2* gene located on chromosome 18. MethPrimer prediction identified a larger CpG island spanning 1350 bp from −694 to +656 bp and a smaller CpG island at the +717 to +835 bp region (Fig. 3*A*). The larger CpG island is densely populated with CpGs distributed across the proximal 5′ upstream region and the first exon, suggesting its potential critical role in regulating *Slc12a2* transcription. MeDIP-qPCR analysis demonstrated that CpGs within three regions of the *Slc12a2* promoter in the PVN were largely unmethylated in 13-week-old SHR compared to age-matched WKY (n = 6 rats per group; Fig. 3*B*). Specifically, the enrichment of 5mC at the upstream region from −490 to −359 bp, which includes 10 CpGs, was significantly reduced in the PVN of SHR compared to WKY (t (10) = 48.42, *p* < 0.001; n = 6 rats per group; Fig. 3, B). Moreover, the enrichment of methylated CpGs at the regions from −72 to +54 bp (with 21 CpGs; t (10) = 47.90, *p* < 0.001) and from +238 to +327 bp (with 11 CpGs; t (10) = 32.28, *p* < 0.001) of the *Slc12a2* promoter in the PVN was similarly reduced in 13-week-old SHR compared to age-matched WKY (Fig. 3*B*).

Furthermore, we determined whether DNA hypomethylation at the *Slc12a2* promoter in the PVN occurs in 4-week-old SHR. MeDIP-qPCR assay showed that DNA methylation levels in the same three regions (−490 to −359 bp, t (10) = 2.446, *p* = 0.034; −72 to +54 bp, t (10) = 8.344, *p* < 0.001; +238 to +327 bp, t (10) = 7.217, *p* < 0.001) of the *Slc12a2* promoter in the PVN displayed a small but statistically significant reduction in 4-week-old male SHR compared to age-matched male WKY (n = 6 rats per group; Fig. 3*C*).

BSP analysis of two cloned regions of the *Slc12a2* promoter in the PVN revealed that 39 CpGs within −370 to −139 bp were largely demethylated in 13-week-old SHR compared to age-matched WKY (Fig. 3*D*). In addition, BSP analysis of another region within the first exon at +320 to +523 bp in the PVN showed that 18 out of 21 CpGs were greatly unmethylated in SHR compared to WKY (Fig. 3*D*). These data collectively indicate that DNA methylation at the *Slc12a2* promoter in the PVN is progressively diminished in SHR.

### Enrichment of DNMT1, DNMT3A, and MeCP2 at *Agtr1a* and *Slc12a2* promoters in the PVN is diminished in SHR

**Figure 4:**
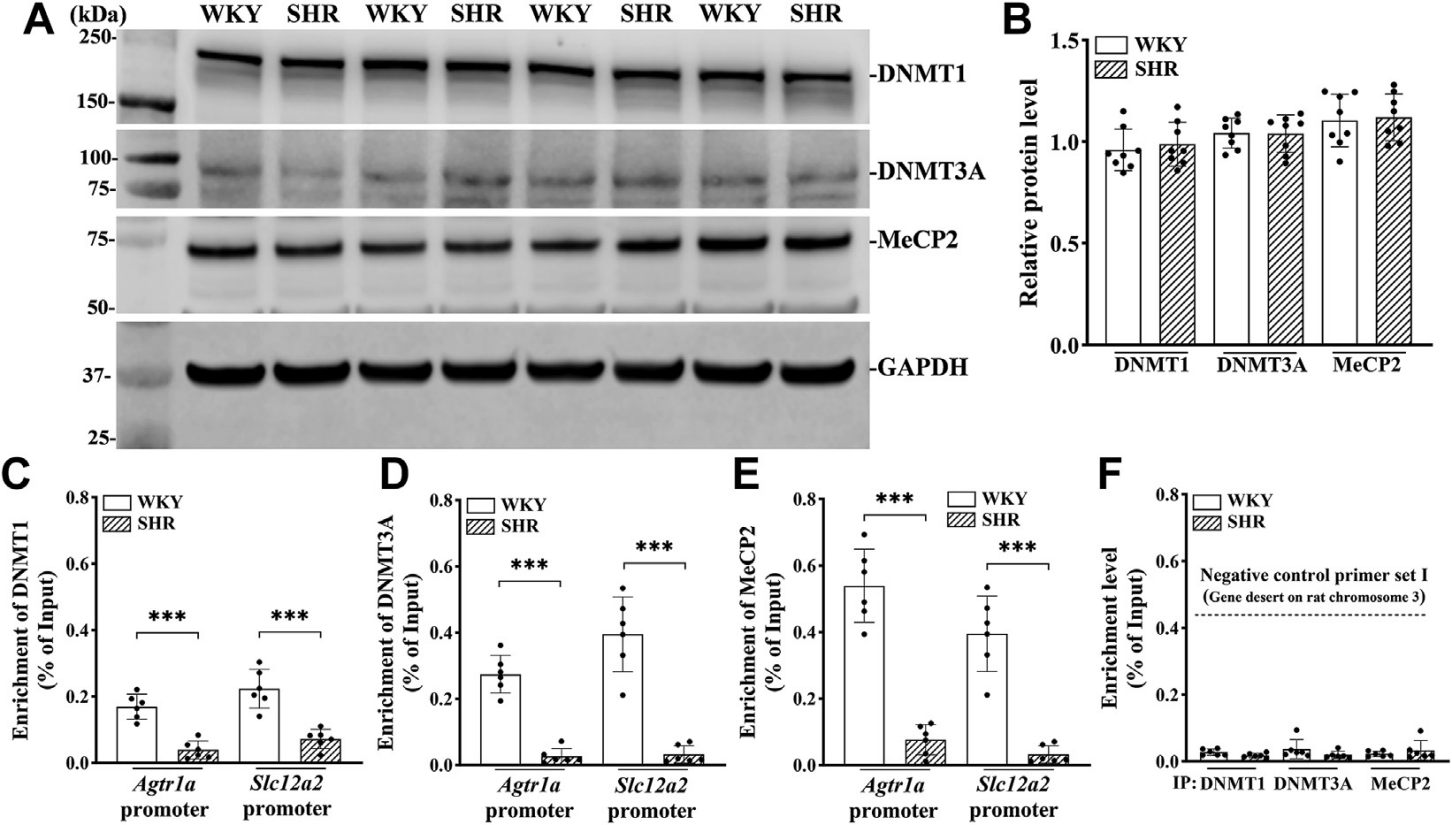
**Reduced enrichment of DNA methylation writer and reader proteins at the *Agtr1a* and *Slc12a2* promoter in the PVN of SHR.***A* and *B*, representative blotting images (*A*) and quantification (*B*) show the total protein levels of DNMT1, DNMT3A, and MeCP2 proteins in the PVN tissues of 13-week-old male SHR and WKY (n = 8 rats per group). GAPDH was used as a loading control. *C*–*E*, chromatin immunoprecipitation-qPCR was performed to quantify the binding amount of the DNMT1 (*C*), DNMT3A (*D*), and MeCP2 (*E*) proteins at the promoter of *Agtr1a* (−9/+137 bp) and *Slc12a2* (−72/+54 bp) (n = 6 rats per group). *F*, qPCR assays of the rat negative control primer set I using chromatin DNA immunoprecipitated with the DNMT1, DNMT3A, and MeCP2 antibodies. Rabbit IgG was used as a negative ChIP control for all immunoprecipitation assays in the above experiment. Data are represented as mean ± SD. ∗∗∗*p* < 0.001 (two-tailed Student’s *t* test). ChIP, chromatin immunoprecipitation; DNMT, DNA methyltransferase; IgG, immunoglobulin G; MeCP2, methyl-CpG binding protein 2; PVN, paraventricular nucleus; qPCR, quantitative PCR; SHR, spontaneously hypertensive rats; WKY, Wistar-Kyoto rats.

DNMT enzymes play a crucial role in catalyzing the methylation of cytosine bases on DNA. DNMT1 acts as a maintenance methyltransferase, primarily targeting hemimethylated palindromic CpGs during DNA replication when cells divide (27). On the other hand, DNMT3A and DNMT3B are considered *de novo* methyltransferases, responsible for adding a methyl group to unmethylated cytosines to establish initial DNA methylation, especially during embryonic development (28). DNMT1 is widely expressed in the central nervous system, including the hypothalamus (29). While DNMT3A is present in both embryonic and postnatal brain tissues, DNMT3B is only detected within a narrow window during early neurogenesis (30, 31). Immunoblotting analysis of PVN tissues showed that the total protein levels of DNMT1 and DNMT3A did not differ significantly between WKY and SHR (n = 8 rats per group; Figure 4, *A* and *B*). However, the DNMT3B protein in the PVN was not detectable in either WKY or SHR.

Measuring global levels of DNMT proteins in PVN tissues cannot predict their abundance locally at the gene promoter. Because the CpGs at the *Agtr1a* and *Slc12a2* promoters in the PVN are largely unmethylated in SHR, we investigated the enrichment of DNMT1 and DNMT3A at these promoters using chromatin immunoprecipitation-qPCR (ChIP-qPCR). The enrichment of DNMT1 at the −9 to +137 bp region of the *Agtr1a* promoter in the PVN was significantly reduced in 13-week-old SHR compared to age-matched WKY (t (10) = 6.937, *p* < 0.001; n = 6 rats per group; Fig. 4*C*). Similarly, the DNMT1 binding at the *Slc12a2* promoter (−72 to +54 bp region) in the PVN was diminished in SHR compared to WKY (t (10) = 5.705, *p* < 0.001; n = 6 rats per group; Fig. 4, C). Interestingly, the enrichment of DNMT3A in the same promoter regions of *Agtr1a* (t (10) = 9.858, *p* < 0.001; n = 6 rats per group; Fig. 4*D*) and *Slc12a2* (t (10) = 7.638, *p* < 0.001; n = 6 rats per group; Fig. 4*D*) in the PVN was also significantly lower in SHR than in WKY. These findings indicate that both *de novo* and maintenance DNMTs are reduced at the hypomethylated promoters of *Agtr1a* and *Slc12a2* in the PVN of SHR.

Methylated DNA often recruits methyl-CpG-binding domain proteins, such as MeCP2, which further aid chromatin remodeling and transcriptional regulation (32, 33). The total protein level of MeCP2 in the PVN did not differ significantly between 13-week-old SHR and age-matched WKY (n = 8 rats per group; Figure 4, *A* and *B*). ChIP-qPCR analysis showed that the binding of MeCP2 at the promoters of *Agtr1a* (t (10) = 9.548, *p* < 0.001) and *Slc12a2* (t (10) = 7.638, *p* < 0.001) in the PVN was profoundly reduced in 13-week-old SHR compared to age-matched WKY (n = 6 rats per group; Fig. 4*E*). The specificity of the antibodies used for ChIP-qPCR was confirmed using commercially available negative control primers (set I) that amplify a gene desert region on rat chromosome 3, which showed no significant DNA amplification from the immunoprecipitated chromatin (n = 6 rats per group; Fig. 4*F*). These data suggest that the hypomethylated promoters of *Agtr1a* and *Slc12a2* in the PVN are associated with diminished DNA methylation writer and reader proteins in SHR.

### Abundance of TETs at *Agtr1a* and *Slc12a2* promoters in the PVN is increased in SHR

**Figure 5:**
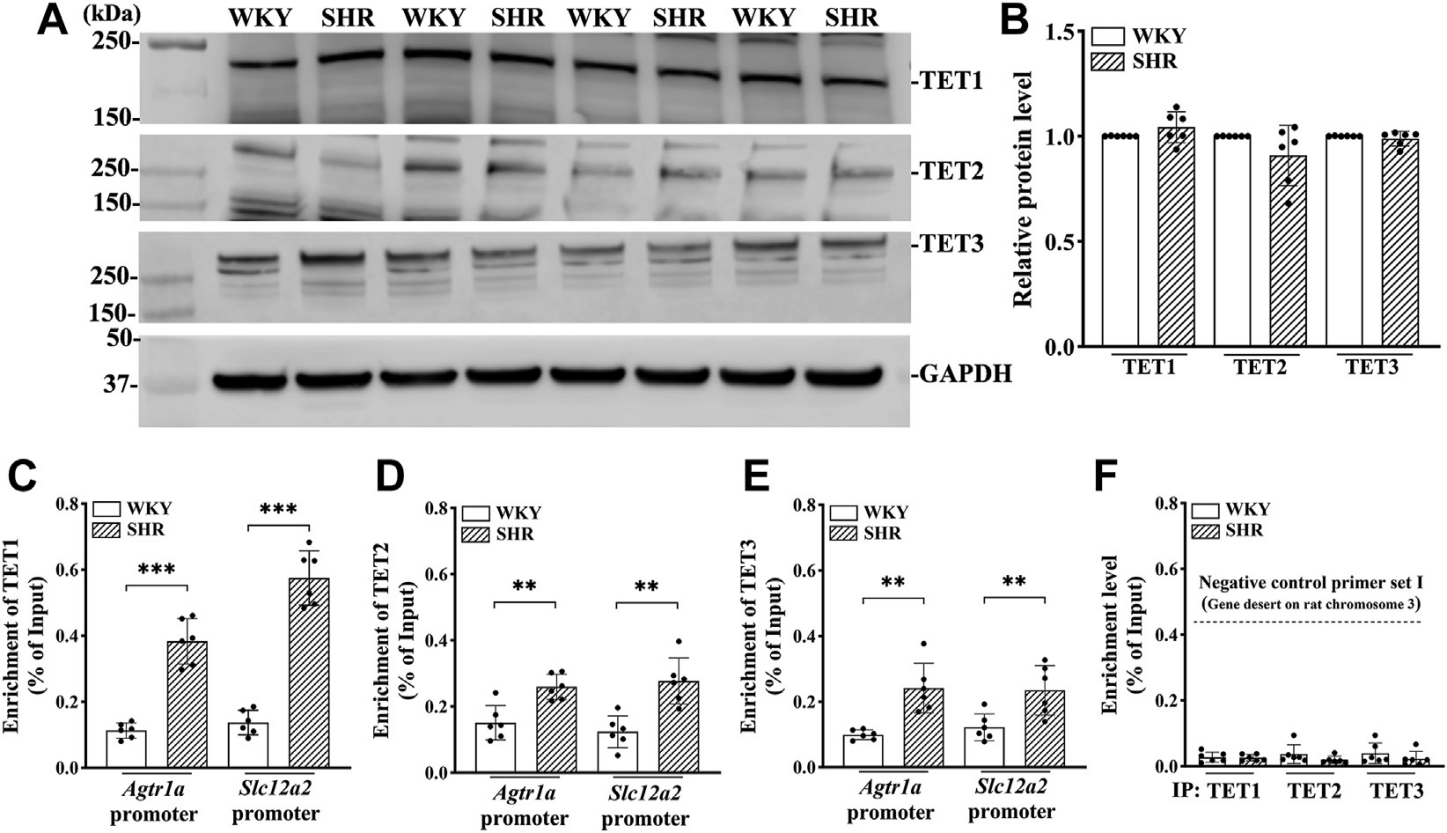
**Increased abundance of DNA methylation eraser proteins at the *Agtr1a* and *Slc12a2* promoter in the PVN of SHR.***A* and *B*, representative blotting images (*A*) and quantification (*B*) show the total protein levels of TET1, TET2, and TET3 in the PVN tissues of 13-week-old male SHR and WKY rats (n = 8 rats per group). GAPDH was used as a loading control. *C*–*E*, chromatin immunoprecipitation-qPCR assay was used to quantify the binding of TET1(*C*), TET2 (*D*), and TET3 (*E*) at the promoter of *Agtr1a* (−9/+137 bp) and *Slc12a2* (−72/+54 bp). (n = 6 rats per group). *F*, qPCR assay was performed with the rat negative control primer set I using the chromatin DNA immunoprecipitated with TET1, TET2, and TET3 antibodies in the above experiment. Rabbit IgG was used as a negative ChIP control for all immunoprecipitation assays in the above experiment. Data are represented as mean ± SD. ∗∗*p* < 0.01, ∗∗∗*p* < 0.001 (two-tailed Student’s *t* test). ChIP, chromatin immunoprecipitation; IgG, immunoglobulin G; PVN, paraventricular nucleus; qPCR, quantitative PCR; SHR, spontaneously hypertensive rats; TET, ten-eleven translocation; WKY, Wistar-Kyoto rats.

Three TET enzymes (TET1, TET2, and TET3) facilitate demethylation of 5mC, aiding reversal from a repressive promoter to an active promoter in gene expression (12). We next determined whether the enrichment of TET3 is altered at promoters of *Agtr1a* and *Slc12a2* in the PVN of SHR. Immunoblotting analysis showed that the total protein levels of TET1, TET2, and TET3 in the PVN did not differ significantly between 13-week-old SHR and age-matched WKY (n = 6 rats per group; Fig. 5, *A* and *B*). However, ChIP-qPCR assays demonstrated a large increase in the enrichment of TET1 in the −9 to +137 bp region of the *Agtr1a* promoter (t (10) = 9.121, *p* < 0.001) and in the −72 to +54 bp region of the *Slc12a2* promoter (t (10) = 11.86, *p* < 0.001) in the PVN of SHR compared to WKY (n = 6 rats per group; Fig. 5*C*). Furthermore, the enrichment of TET2 in the same region of the *Agtr1a* promoter (t (10) = 4.111, *p* = 0.002) and the *Slc12a2* promoter (t (10) = 4.4437, *p* = 0.0013) in the PVN was significantly increased in SHR compared to WKY (n = 6 rats per group, Fig. 5*D*). Additionally, the binding of TET3 at the *Agtr1a* promoter (t (10) = 4.530, *p* = 0.0011) and the *Slc12a2* promoter (t (10) = 3.207, *p* = 0.0094) in the PVN was significantly greater in SHR compared to WKY (n = 6 rats per group, Fig. 5*E*). The specificity of the TET antibodies used for ChIP-qPCR was again confirmed by showing no significant enrichment at a gene-desert (n = 6 rats per group; Fig. 5*F*). These findings indicate a heightened presence of TETs at the promoters of *Agtr1a* and *Slc12a2*, which may induce active DNA demethylation of these promoters in the PVN of SHR.

### DNMT inhibition in the PVN increases *Agtr1a* and *Slc12a2* transcription and ABP in WKY

**Figure 6:**
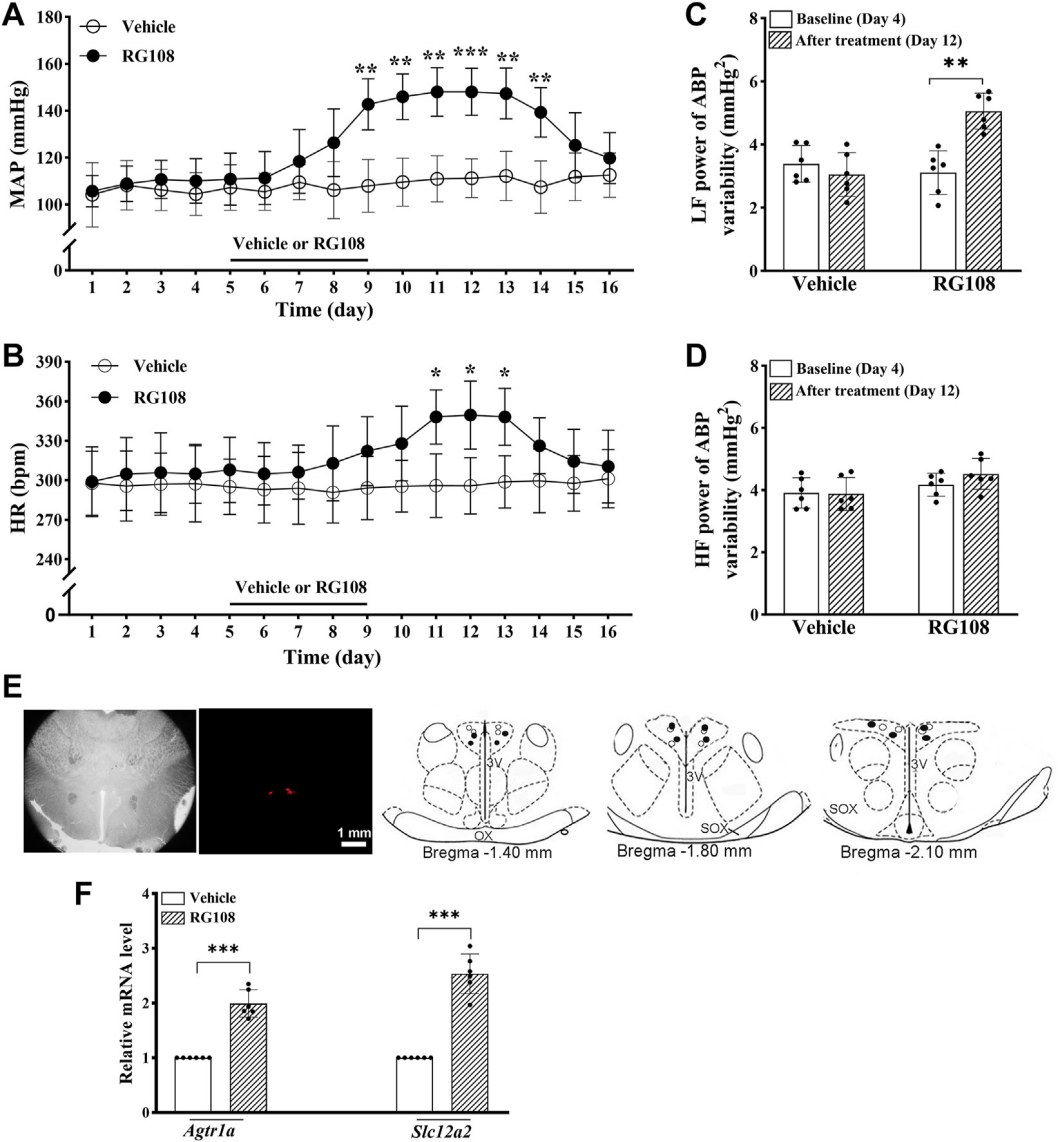
**DNMT inhibition in the PVN increases arterial blood pressure and transcription of *Agtr1a* and *Slc12a2* in normotensive WKY.***A* and *B*, time course of changes in mean arterial blood pressure (MAP, *A*) and heart rate (HR, *B*) measured using telemetry in conscious 13-week-old male WKY microinjected with RG108 (25 μg/50 nl per day) or vehicle (0.05% DMSO) into the PVN for 5 days (n = 6 rats per group). ∗*p* < 0.05, ∗∗*p* < 0.01, ∗∗∗*p* < 0.001, RG108 *versus* the vehicle group at the same time point. *C* and *D*, the low-frequency (LF, *C*) and high-frequency (HF, *D*) power of systolic ABP variability in WKY microinjected with vehicle or RG108 into the PVN (n = 6 rats per group). ∗∗*p* < 0.01. *E*, representative brightfield and fluorescence images and schematic drawing show the microinjection sites in the PVN of WKY at three levels (vehicle, *empty circles*; RG108, *filled circles*). 3V, third ventricle, OX, optic chiasm; SOX, supraoptic decussation. *F*, the mRNA levels of the *Agtr1a* and *Slc12a2* in the PVN, measured using qPCR and normalized to *Gapdh*, in vehicle- and RG108-treated WKY (n = 6 rats per group). ∗∗∗*p* < 0.001. Data are represented as mean ± SD (two-way ANOVA followed by Bonferroni's *post hoc* test in (*A–D*); two-tailed Student’s *t* test in *F*). ABP, arterial blood pressure; DNMTs, DNA methyltransferases; DMSO, dimethyl sulfoxide; PVN, paraventricular nucleus; qPCR, quantitative PCR; WKY, Wistar-Kyoto rats.

Our experiments above showed an association between DNA hypomethylation and diminished DNMT binding at the *Agtr1a* and *Slc12a2* promoters in the PVN in SHR. To determine the functional significance of DNMTs in the PVN in regulating the expression of *Agtr1a* and *Slc12a2* as well as ABP in normotensive WKY, we conducted microinjections of N-phthalyl-L-tryptophan (RG108), a small-molecule DNMT inhibitor, or vehicle into the PVN of 13-week-old WKY. RG108 specifically inhibits DNA methyltransferases, leading to DNA demethylation and activation of methylation-silenced genes (24, 34). Bilateral microinjection of 25 μg (in 50 nl) of RG108, but not vehicle, into the PVN for five consecutive days caused a time-dependent increase in mean ABP and heart rate (HR) (n = 6 rats per group; Fig. 6, *A*–*E*). The elevated mean ABP level lasted about 5 days after discontinuing RG108 treatment.

Because long-term stable recording of sympathetic nerve activity in conscious animals is very difficult, we analyzed the low-frequency component of the power spectrum of systolic ABP variability, which could be used as an index of sympathetic outflow in conscious states (35, 36). The low- and high-frequency power of systolic ABP variability did not differ significantly between the vehicle and RG108 groups at baselines (n = 6 rats per group; Fig. 6, *C* and *D*). Bilateral microinjection of RG108, but not vehicle, into the PVN of WKY for 5 days significantly increased the low-frequency power of systolic ABP variability (F_(1.10)_ = 17.34, *p* = 0.0011; Fig. 6*C*). However, treatment with RG108 had no significant effect on the high-frequency power of systolic ABP variability in WKY (Fig. 6*D*). In addition, RG108 treatment for 5 days caused a substantial increase in the mRNA levels of *Agtr1a* (t (10) = 9.690, *p* < 0.001) and *Slc12a2* (t (10) = 10.36, *p* < 0.001) in the PVN (n = 6 rats per group; Fig. 6*F*). These results suggest that intrinsic DNMT activity in the PVN has a significant role in regulating ABP, sympathetic vasomotor tone, and transcription of *Agtr1a* and *Slc12a2*.

### TET inhibition in the PVN attenuates elevated ABP and transcription of *Agtr1a* and *Slc12a2* in SHR

**Figure 7:**
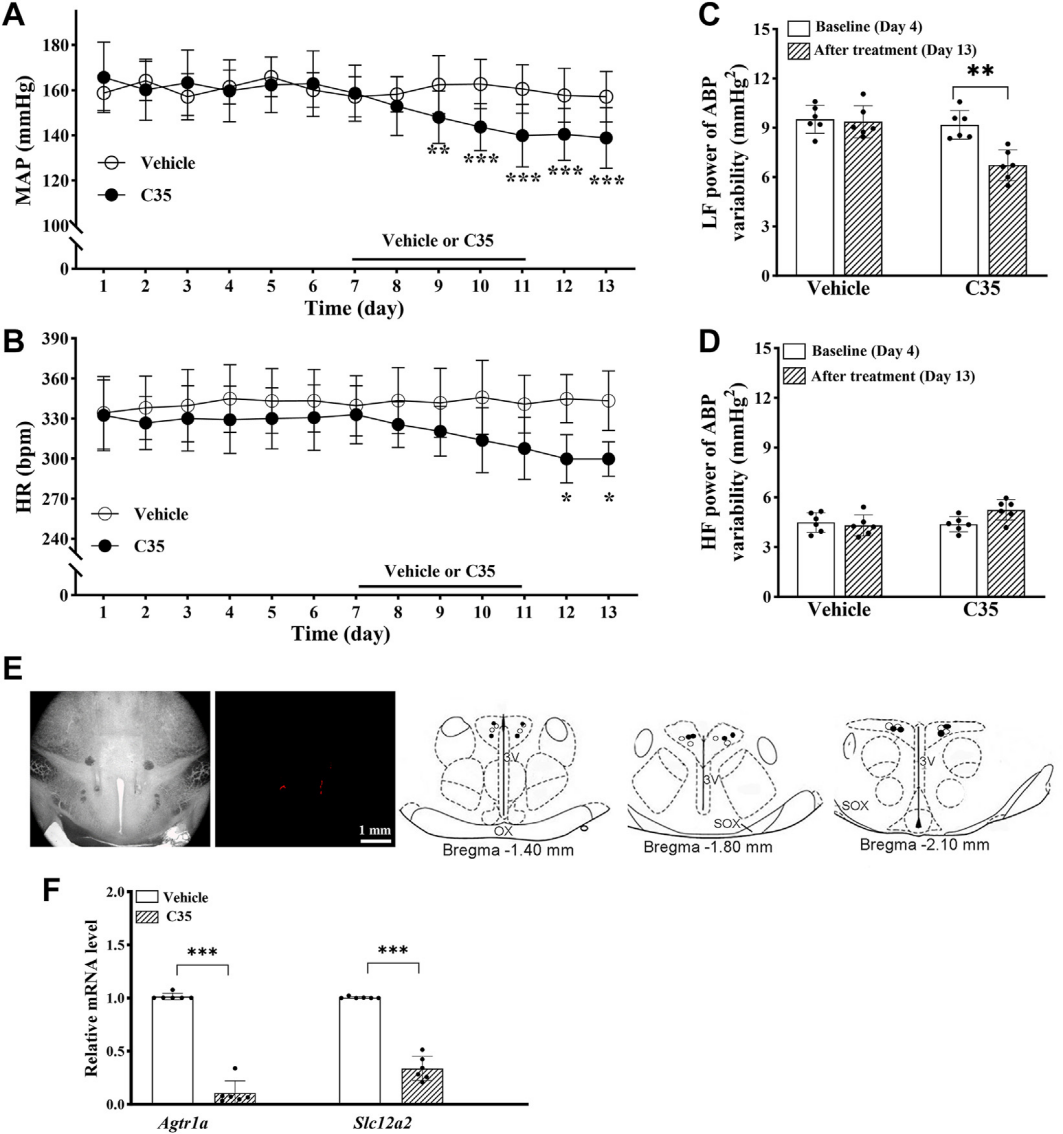
**TET inhibition in the PVN attenuates elevated arterial blood pressure and transcription of *Agtr1a* and *Slc12a2* in SHR.***A* and *B*, time course of changes in mean arterial blood pressure (MAP, *A*) and heart rate (HR, *B*) measured using telemetry in conscious 13-week-old male SHR microinjected with C35 (10 μg/50 nl per day) or vehicle (0.05% DMSO) into the PVN for 5 days (n = 6 rats per group). ∗*p* < 0.05, ∗∗*p* < 0.01, ∗∗∗*p* < 0.001, C35 *versus* the vehicle group at the same time point. *C* and *D*, the low-frequency (LF, *C*) and high-frequency (HF, *D*) power of systolic ABP variability in SHR microinjected with vehicle or C35 into the PVN (n = 6 rats per group). ∗∗*p* < 0.01. *E*, representative brightfield and fluorescence images and schematic drawing show the microinjection sites in the PVN of SHR at three levels (vehicle, *empty circles*; C35, *filled circles*). 3V, third ventricle, OX, optic chiasm; SOX, supraoptic decussation. *F*, the mRNA levels of the *Agtr1a* and *Slc12a2* in the PVN, measured using qPCR and normalized to *Gapdh*, in vehicle- and C35-treated SHR (n = 6 rats per group). ∗∗∗*p* < 0.001. Data are represented as mean ± SD (two-way ANOVA followed by Bonferroni's *post hoc* test in *A–D*; two-tailed Student’s *t* test in *F*). ABP, arterial blood pressure; DMSO, dimethyl sulfoxide; PVN, paraventricular nucleus; qPCR, quantitative PCR; SHR, spontaneously hypertensive rats.

Finally, because DNA hypomethylation is also associated with increased TET enrichment at the promoters of *Agtr1a* and *Slc12a2*, we determined whether TET activity in the PVN controls the transcription of *Agtr1a* and *Slc12a2* as well as ABP in SHR. To this end, we microinjected TET-IN-C35 (C35), a specific TET inhibitor (37), or vehicle into the PVN of 13-week-old SHR. Bilateral microinjection of 10 μg (in 50 nl) of C35, but not vehicle, into the PVN for five consecutive days gradually and significantly reduced mean ABP and HR in SHR (n = 6 rats per group; Fig. 7, *A*–*E*). These decreased levels of mean ABP and HR lasted at least an additional 2 days after discontinuing C35 treatment.

Power spectrum analysis showed that the low- and high-frequency power of systolic ABP variability was similar between the vehicle and C35 groups at baselines (n = 6 rats per group; Fig. 7, *C* and *D*). Bilateral microinjection of C35, but not vehicle, into the PVN of SHR for 5 days significantly reduced the low-frequency power of systolic ABP variability (F_(1.10)_ = 9.10, *p* = 0.0021; Fig. 7*C*). However, treatment with C35 had no significant effect on the high-frequency power of systolic ABP variability in SHR (Fig. 7*D*). Furthermore, the mRNA level of *Agtr1a* (t(10) = 18.66, *p* < 0.001) and *Slc12a2* (t(10) = 14.28, *p* < 0.001) in the PVN was much lower in C35-injected SHR than in vehicle-injected SHR (n = 6 rats per group; Fig. 7*F*). These data suggest that the elevated ABP, sympathetic outflow, and increased transcription of *Agtr1a* and *Slc12a2* are sustained by TET activity in the PVN of SHR.

## Discussion

Our study demonstrates an age-dependent increase in the transcripts of *Agtr1a* and *Slc12a2*, two critically important genes in the PVN involved in the development of hypertension. The PVN is a major source of increased sympathetic vasomotor activity in hypertension (3, 6, 7). *Agtr1a* knockdown in the PVN prevents salt-sensitive hypertension in mRen-2 rats (38). AT_1A_, but not AT_1B_, receptors are expressed in the PVN (39), and AT_1A_ receptor expression in the PVN is much higher in SHR than in WKY (19, 40). The transcriptional changes of *Agtr1a* and *Slc12a2* correlate with the functional significance and protein levels of the angiotensin AT_1A_ receptor and NKCC1 in the PVN in SHR. In this regard, AT_1A_ receptor stimulation impairs GABAergic input but enhances glutamatergic input to PVN presympathetic neurons, leading to an elevated sympathetic outflow (19, 41, 42). Additionally, upregulation of NKCC1 in the PVN elevates intracellular chloride level, reducing GABAergic synaptic inhibition of PVN presympathetic neurons and contributing to increased sympathetic drive in SHR (22). In the present study, we focused on changes in the mRNA level of AT_1A_ receptors and NKCC1 in PVN tissues from WKY and SHR at prehypertensive (4-week-old), early hypertensive (7-week-old), and established hypertensive (13-week-old) stages. Although their expression level in the PVN was similar between 4-week-old WKY and SHR, the mRNA levels of *Agtr1a* and *Slc12a2* were markedly increased in the PVN when hypertension occurred in SHR, indicating that progressive upregulation of AT_1A_ receptors and NKCC1 in the PVN is associated with hypertension development.

Our findings reveal profound DNA hypomethylation at the promoters of *Agtr1a* or *Slc12a2* in the PVN of SHR. DNA methylation, specifically CpG methylation, is an inheritable epigenetic mark that regulates gene transcriptional in a stable yet reversible manner (23). CpG dinucleotides are highly concentrated within CpG islands at the gene promoters and remain unmethylated to promote transcription (43). The methylated CpGs within the regulatory regions, such as enhancers and promoters, along with the first exon can suppress gene transcription, whereas hypomethylation promotes gene transcription (44). Methylated CpGs can hinder transcription machineries (*e.g.*, transcription factors and cofactors) to access DNA or recruit large repressor protein complexes, leading to gene silencing (45). Using two complementary approaches to examine the DNA methylation status, we found that CpGs at the *Agtr1a* and *Slc12a2* promoters in the PVN were unmethylated in adult SHR. Furthermore, a substantial number of CpGs located throughout the first exon proximal to the TSS of *Agtr1a* and *Slc12a2* was unmethylated in the PVN of SHR. Although the 5′ upstream regions near the first exon of *Agtr1a* have fewer CpGs, the downstream exon and following intronic sequence are CpG-rich. The hypomethylation of these CpGs allows for active transcription of *Agtr1a* in the PVN of SHR. Similarly, the *Slc12a2* promoter, especially the region surrounding the TSS, exhibits high CpG density, and the majority of these CpGs undergo demethylation, leading to the transcriptional activation of *Slc12a2* in the PVN of SHR. We found that DNA methylation levels at *Agtr1a* and *Slc12a2* promoters in the PVN were also significantly decreased in 4-week-old normotensive SHR, although this decrease is comparatively less pronounced than the reduction observed in 13-week-old SHR. These findings suggest that progressive DNA demethylation likely plays a significant role in the “priming” of transcriptional activation of *Agtr1a* and *Slc12a2*, contributing to the onset of hypertension.

A striking finding of our study is the switch in the enrichment of DNMTs and TETs at the hypomethylated promoters of *Agtr1a* and *Slc12a2* in the PVN of SHR. The DNA methylation status at a gene promoter depends on the balance between DNMT and TET enzymes’ catalytic activities, along with the recognition of methylated CpGs by reader proteins such as MeCP2 (46, 47). DNA methylation is transgenerationally inheritable, with DNMT1 mainly responsible for maintaining methylation on hemimethylated DNA strands (48). But DNMT3 enzymes are mostly involved in *de novo* methylation of unmethylated DNA strands (49). Our study reveals a significant reduction in the binding of DNMT1 and DNMT3A at the promoter of *Agtr1a* and *Slc12a2* in the PVN of SHR. DNMT3B and MeCP2 are associated with DNA hypomethylation at the *Slc12a2* promoter in mesenteric arteries of adult SHR (50). Furthermore, offspring of pregnant mother rats exposed to dexamethasone or low-protein diet exhibit increased *Agtr1a* expression and salt-induced hypertension, which is associated with reduced DNMT3A binding at the *Agtr1a* promoter in the PVN (51). In our study, we found that both DNMT1 and DNMT3A were disassociated from a similar region of *Agtr1a* and *Slc12a2* promoters in the PVN of SHR.

Our findings suggest a potential role of TET-mediated active demethylation of CpGs at the *Agtr1a* and *Slc12a2* promoters, which may contribute to their transcriptional activation in the PVN of SHR. We showed that all three TET enzymes, particularly TET1, were markedly enriched at promoters of *Agtr1a* and *Slc12a2* in the PVN of SHR. Enhanced binding of TETs to these promoters could shift the balance toward an active demethylation of CpGs at the gene’s regulatory regions, facilitating transcriptional activation (12). Among the three TET proteins, TET1 primarily recognizes and binds to CpG-rich DNA sequences through its N-terminal CXXC-domain, whereas the core catalytic domain at the C-terminal induces oxidation of 5mC to 5hmC, initiating further DNA demethylation (12, 52). Additionally, the enrichment of TET1's catalytic product, 5hmC, at a gene promoter could facilitate transcriptional regulation (53). The other two TETs, TET2 and TET3, catalyze further oxidation, leading to the formation of 5-formylcytosine and 5-carboxylcytosine, respectively. These modifications ultimately associate with the base excision repair pathway, which is mediated by thymine DNA glycosylase, resulting in the restoration of the unmethylated cytosine state (54). The enrichment of DNMTs may be replaced by that of TETs at the promoters of *Agtr1a* and *Slc12a2* in the PVN of adult SHR, and this chromatin plasticity favors active DNA demethylation and transcriptional activation of these genes. Further studies are needed to understand the mechanisms underlying the distinct switch in the enrichment of DNMTs and TETs at these gene promoters in SHR.

In addition to the switch of DNMT/TET bindings at the *Agtr1a* and *Slc12a2* promoters, we found a substantial reduction in the enrichment of MeCP2 at these two hypomethylated promoters in the PVN of SHR. Methylated cytosines at the regulatory regions recruit methyl-CpG binding domain proteins, such as MeCP2, which can read the methylated DNA and influence the accessibility of transcription factors to the DNA (14). MeCP2 is highly expressed in neurons and primarily acts to restrain gene transcription, and the prevalent MeCP2 binding at gene promoters relies on the presence of CpG methylation (55). The demethylation of the *Agtr1a* and *Slc12a2* promoters likely reduces the recruitment of MeCP2, facilitating the active transcription of these genes in the PVN of SHR. It is unclear how changes in the enrichment of DNMTs, TETs, and MeCP2 at gene promoters are orchestrated to synergistically promote the transcription of *Agtr1a* and *Slc12a2* in the PVN of SHR. Further studies are necessary to uncover the epigenetic landscapes and define functional crosstalk between DNA methylation regulators and MeCP2 at the promoters of hypertension-related genes in the brain of SHR.

Another salient finding of our study is that the DNA methylation status in the PVN directly controls *Agtr1a* and *Slc12a2* expression and ABP levels. We showed that inhibition of DNMT activity with RG108 in the PVN increased expression levels of *Agtr1a* and *Slc12a2*, along with a concurrent elevation in ABP in normotensive rats. Conversely, inhibition of TET activity with C35 in the PVN led to decreased expression of *Agtr1a* and *Slc12a2*, accompanied by a reduction in ABP in SHR. Unlike other DNMT inhibitors such as 5-Aza-dCR, RG108 is a nonnucleoside analog and can directly bind to DNMTs and interfere with their activity, leading to DNA hypomethylation (34). The TET inhibitor C35, a phylloflavan molecule, blocks the catalytic activity of TET enzymes, resulting in the reduction of the 5hmC marks throughout the genome and enabling the removal of methyl groups from cytosine residues (37). The decreased expression of *Agtr1a* and *Slc12a2* in the PVN upon TET inhibition likely restores the balance of glutamatergic excitatory and GABAergic inhibitory input to PVN presympathetic neurons, thereby reducing sympathetic vasomotor activity in SHR. Our findings strongly indicate a cause-and-effect relationship between dynamic DNA methylation, jointly controlled by DNMTs and TETs, and the altered transcription of *Agtr1a* and *Slc12a2* in the PVN, which ultimately influences ABP. Nevertheless, inhibition of DNMTs or TETs in the PVN may directly or indirectly affect the expression of many other genes, such as *Grm5* and *Cacna2d1*, which also contribute to elevated sympathetic outflow in SHR (9, 10).

**Figure 8:**
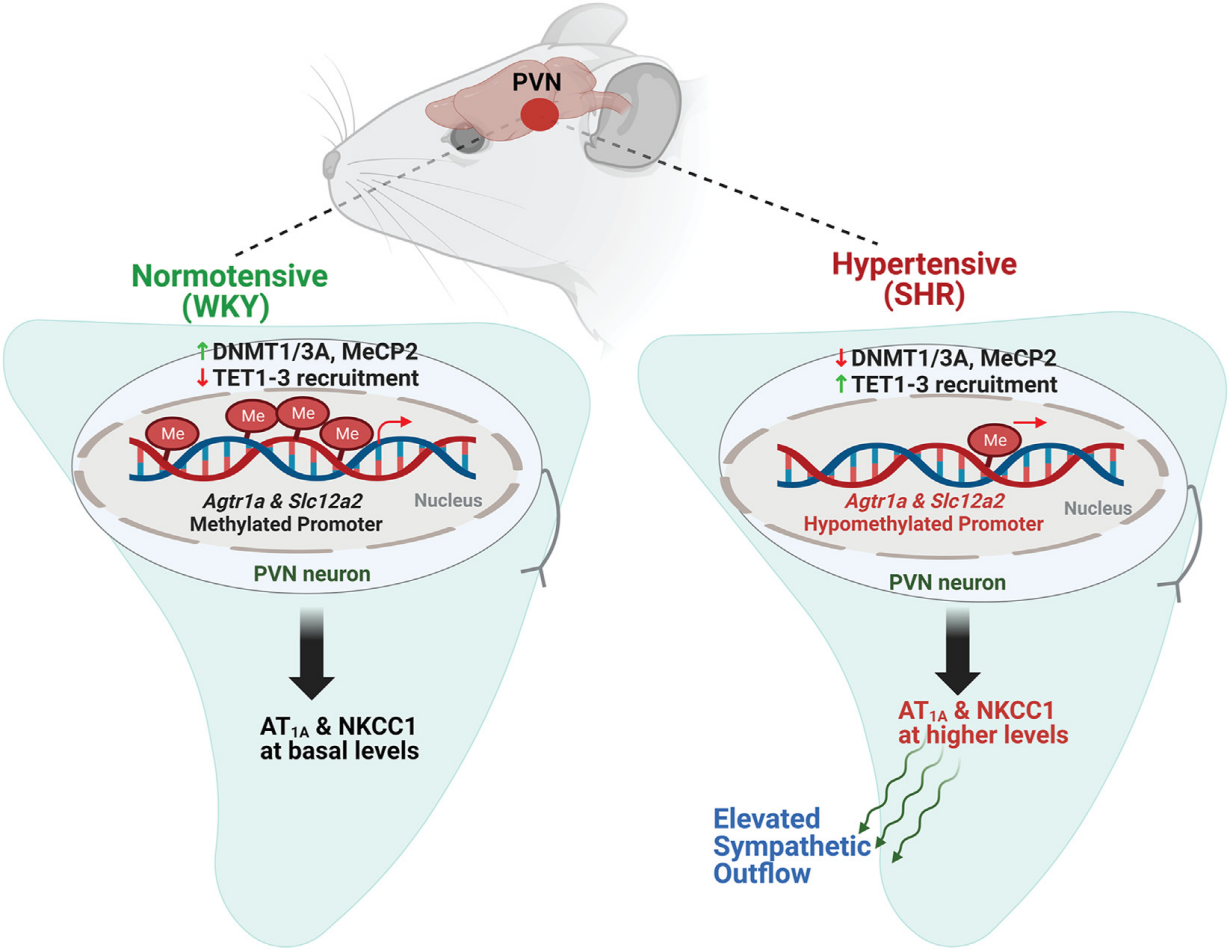
**Schematic representation shows the relationship between DNA methylation and transcription of *Agtr1a* and *Slc12a2* in the PVN**. In the PVN of normotensive WKY, DNMTs, and MeCP2 are highly enriched, whereas the presence of TETs is low, at the promoters of *Agtr1a* and *Slc12a2*, restricting their transcription. By contrast, in the PVN of SHR, the enrichment of DNMTs and MeCP2 is diminished, whereas the abundance of TETs is increased, resulting in DNA demethylation at *Agtr1a* and *Slc12a2* promoters and transcriptional activation of these genes. Consequently, upregulation of AT_1A_ receptors and NKCC1 in the PVN could augment sympathetic outflow and contribute to the development of hypertension. DNMTs, DNA methyltransferase; MeCP2, methyl-CpG binding protein 2; NKCC1, Na^+^-K^+^-Cl^−^ cotransporter-1; PVN, paraventricular nucleus; SHR, spontaneously hypertensive rats; TET, ten-eleven translocation; WKY, Wistar-Kyoto rats.

In summary, our study unveils that DNA hypomethylation plays a crucial role in active transcription of *Agtr1a* and *Slc12a2*, two major prohypertensive genes in the brain, in the development of hypertension. The diminished enrichment of DNMTs and MeCP2, coupled with the heightened abundance of TETs, at *Agtr1a* and *Slc12a2* promoters creates a permissive epigenetic landscape that actively drives the transcription of *Agtr1a* and *Slc12a2* in the PVN (Fig. 8), contributing to the development of hypertension. The activity of DNMTs or TETs in the PVN directly influences ABP, likely through regulating *Agtr1a* and *Slc12a2* transcription. These findings advance our understanding of epigenetic programming occurring in the brain during the development of hypertension. Restoring DNA methylation of hypertension-promoting genes may have a long-lasting effect on the control of hypertension.

## Experimental procedures

### Animals

All experimental protocols were approved by the Institutional Animal Care and Use Committee of The University of Texas MD Anderson Cancer Center and performed in compliance with the guidelines on the ethical use of animals of National Institutes of Health. Male and female WKY and SHR (4–13 weeks old) were obtained from Envigo. Animals were housed (3 rats per cage) on a 12 h light/dark cycle and provided with *ad libitum* access to food and water.

### PVN cannulation and microinjection

Rats were anesthetized using 3% isoflurane and positioned on a stereotaxic apparatus. A bilateral guide cannula (26-gauge with a 1.0 mm interval between two barrels, extending 7.0 mm from the pedestal; RWD Life Science Inc) was implanted into the rat PVN based on the following stereotactic coordinates: 1.4 to 2.1 mm caudal to the bregma, 0.1 to 0.5 mm lateral to the midline, and 7.0 to 7.5 mm ventral to the dura (8, 19). The tip of the guide cannula was positioned 1.0 mm dorsal to the bilateral sites of the PVN. The guide cannula was secured to the skull using dental acrylic. A dummy cannula was then inserted into the two barrels of the guide cannula, and a dust cap was placed over the external end of the dummy cannula. After the surgery, the rats received subcutaneous injection of an antibiotic (5 mg/kg enrofloxacin, daily for 3 days) and an analgesic (0.5 mg/kg buprenorphine, every 12 h for 2 days). Following a 1-week recovery period, the dummy cannula was removed, and a bilateral needle with tips protruding 1.0 mm beyond the guide cannula tip was used for microinjection. The injection site was confirmed by including fluorescent microspheres (0.04 μm; Invitrogen) in the microinjection solution and plotted according to the rat brain atlas (56).

### Blood pressure measurement with telemetry and power spectrum analysis

A Millar telemetry system was used to continuously monitor and record ABP in freely moving rats. The surgical procedures were similar to the method described previously (57, 58, 59). In brief, rats were anesthetized with 3% isoflurane, and a catheter of telemetry transmitter was inserted into the descending aorta. The transmitter was sutured to the peritoneum. After surgery, the rats were given subcutaneous injection of enrofloxacin (5 mg/kg) daily for 3 days and buprenorphine (0.5 mg/kg) every 12 h for 2 days. The ABP signals were recorded and analyzed using a data acquisition system (LabChart 7; AD Instruments). HR values were derived from ABP pulse signals.

The power spectral analysis (Welch method; 512 points, 50% overlap, and Hamming window) of systolic ABP variability was performed using LabChart 7 (https://www.adinstruments.com/support/downloads/windows/labchart-0). The power spectral density was integrated in the low-frequency range of 0.20–0.75 Hz and high-frequency range of 0.75–3.00 Hz) (60, 61).

### Brain tissue collection

Rat brain tissues, including the PVN, frontal cortex, rostral ventrolateral medulla, and hippocampus, were obtained as we described previously (15, 19, 57, 59). Briefly, rats were quickly decapitated under 3% isoflurane anesthesia, and their brains were immediately removed and placed into ice-cold, 95% O_2_/5% CO_2_-saturated artificial cerebral spinal fluid containing (in mM) 124.0 NaCl, 3.0 KCl, 1.3 MgSO_4_, 2.4 CaCl_2_, 1.4 NaH_2_PO_4_, 10.0 glucose, and 26.0 NaHCO_3_ (300 mOsm). For PVN tissue harvesting, a brain tissue block containing the hypothalamus was glued onto the stage of a vibrating microtome. Coronal hypothalamic slices (400 μm thick) were sectioned, and the PVN was micropunched bilaterally with a slice puncher (0.5 mm diameter) (10, 22).

### Quantitative PCR (qPCR)

Total RNA from the brain tissues was extracted using the RNeasy Plus Universal Mini Kit (#73404; Qiagen), including a quick nonenzymatic removal step of genomic DNA. The purified total RNA was then reverse transcribed to complementary DNA by using the SuperScript IV VILO Master Mix with ezDNase enzyme (#11766050; Invitrogen). A 100 ng of complementary DNA was used for the real-time PCR using QuantStudio 7 Flex Real-Time PCR System (Applied Biosystems) with the PowerUp SYBR Green Master Mix (#A25776; Thermo Fisher Scientific Inc). The fast-cycling mode conditions (50 °C for 2 min, 95 °C for 2 min, 40 cycles of 95 °C for 1 s, and 60 °C for 30 s) were used to perform the quantitative reverse transcription polymerase chain reaction reactions. The qPCR amplicon specificity of each primer pair (Table 1) was verified by melting curve analysis and resolving products by agarose gel electrophoresis. All samples were analyzed in triplicates using the 2^-ΔΔCT^ method, and the mRNA level of target genes was normalized to that of *Gapdh*, the housekeeping gene, in the same sample.

**Table 1:** List of primers used in qPCR

Primer name	Sequence (5′→3′)	Amplicon size (exon-exon junction)
*Agtr1a* forward	TCTCAGCTCTGCCACATTCC	121 bp (Exon 1–3)
*Agtr1a* reverse	CGAAATCCACTTGACCTGGTG	
*Slc12a2* forward	CATGGTGTCAGGATTTGCAC	120 bp (Exon 11–12)
*Slc12a2* reverse	GATATTGTCCTTACATAGAG	
*Gapdh* forward	AGAATGGGAAGCTGGTCATC	111 bp (Exon 3–4)
*Gapdh* reverse	CAGTAGACTCCACGACATACTC	

### MeDIP-qPCR

Methylated DNA immunoprecipitation (MeDIP) was performed according to the method described previously (62). A MeDIP kit (#55009, Active Motif) was used to assess the level of methylated DNA in the tissue sample. In brief, genomic DNA was extracted from the PVN tissues using the DNeasy Blood & Tissue Kit (#69504, Qiagen) according to the manufacturer's instruction. The genomic DNA was fragmented by sonication to achieve fragments of 400 to 800 bp. The fragmented DNA was used for input (10%) and overnight immunoprecipitation using 2 μg of either mouse IgG (the negative control) or mouse anti–5mC antibody in the presence of the bridging antibody (2 μg) and protease inhibitor cocktail provided in the MeDIP kit. The immunoprecipitated DNA was purified using the QIAquick PCR Purification Kit (#28104, Qiagen). The purified DNA was used for MeDIP-qPCR using the specific primers (Table 2) to amplify the promoter DNA regions close to the TSS of *Agtr1a*, *Slc12a2*, and *Grm5* genes. The *Grm5* promoter served as the negative control, as its promoter DNA does not contain any CpG islands within the −1000/+1000 bp region, as predicted by MethPrimer. To determine the relative enrichment of methylated DNA within each target region of the promoter following MeDIP using the 5mC antibody, the percentages were normalized to those in the input sample. The qPCR primer pairs selected for this study efficiently amplify the chosen regions regardless of the methylation status of the CpGs within the amplicons.

**Table 2:** List of primers used in bisulfite sequencing-PCR

Primer name	Sequence (5′→3′)	Amplicon size (number of CpGs)
*Agtr1a* promoter		
−301/–24 bp forward	TGAGTTAGTTATAGTAAAGATAAGGGAG	278 bp (9 CpGs)
−301/–24 bp reverse	TCCCAACTACCTAAAAATCAACAAC	
−85/+142 bp forward	AGTATTTATTTTGGAGTTAGTTTATGTG	227 bp (11 CpGs)
−85/+142 bp reverse	ACTAAACACTTCCATATTTATAACCTAA	
+121/+408 bp forward	TAGGTTATAAATATGGAAGTGTTTAGTT	289 bp (14 CpGs)
+121/+408 bp reverse	TAAAACTCACCAAAAAATATAACAAAAC	
*Slc12a2* promoter		
−370/–139 bp forward	GAGAGGAGTTTATAGGGTT	232 bp (39 CpGs)
−370/–139 bp reverse	AACCCTACGCTAACCAACCTC	
+320/+523 bp forward	GGTAAATTTTTGGGGTTTATTTTTAGT	204 bp (21 CpGs)
+320/+523 bp reverse	AAAACGACCCTTAACTTCCTC	

### Bisulfite sequencing

The MethPrimer program (63) was utilized to predict the presence of CpG islands within a 1000 bp region upstream and downstream of the start site of the first exon sequence, as obtained from the NCBI gene database. The same program was used to design primers for BSP analysis. BSP was performed following previously described methods (62, 64) to investigate the methylation status of individual CpG dinucleotides at the promoters of *Agtr1a* and *Slc12a2*. In brief, genomic DNA from PVN tissues was extracted as described earlier and treated immediately with sodium bisulfite solution using the EpiTect Fast LyseAll Bisulfite Kit (#59864, Qiagen). The converted DNA was then purified and used as a template for amplifying target regions of interest in the genes, using specific primers (Table 3) designed with the MethPrimer tool and EpiMark Hot Start Taq DNA Polymerase (#M0490S, New England Biolabs). The resulting amplicons were cloned into One Shot TOP10 cells using the TOPO TA Cloning Kit for Sequencing (#K457501, Invitrogen). Plasmids were isolated from six individual bacterial colonies and subjected to Sanger DNA sequencing. The DNA sequences from each amplicon were aligned to the Rat genome (mRatBN7.2) for analyzing the methylated and unmethylated CpGs using QUMA, a quantification tool for methylation analysis (65). The experiment was repeated independently at least twice using different sets of samples.

**Table 3:** List of primers used in MeDIP-qPCR

Primer name	Sequence (5′→3′)	Amplicon size (number of CpGs)
*Agtr1a* promoter		
−260/–116 bp forward	CTCCCTCCATCTTCAACACTTC	145 bp (4 CpGs)
−260/–116 bp reverse	CCTGAGACCCTCTGTCCAAC	
−9/+137 bp forward	CAGTTGGGAGGGACTGGATG	146 bp (9 CpGs)
−9/+137 bp reverse	GGACTCACCAGGGAATGTG	
+187/+385 bp forward	TCCTTGTCACCACATCTGAATC	199 bp (9 CpGs)
+187/+385 bp reverse	ACCCTGTACTCGAAAGAGTTTG	
*Slc12a2* promoter		
−490/–359 bp forward	GCGCCCTAAGGGAAACC	132 bp (10 CpGs)
−490/–359 bp reverse	TGGACTCCTCTCGCTTCTT	
−72/+54 bp forward	CGCCGCGCCTTTAAACC	126 bp (21 CpGs)
−72/+54 bp reverse	GGCGCTCCCACTAAGGA	
+238/+327 bp forward	CGGTCGAGAGGATGCTACC	90 bp (11 CpGs)
+238/+327 bp reverse	GGTTTGCCCAGCCCATC	
*Grm5* promoter		
−218/–116 bp forward	GCACTGCCACTTGGAATAAAG	100 bp (2 CpGs)
−218/–116 bp reverse	ACTCCCTGCCATTAACTTCTG	
+215/+320 bp forward	TGACTCAACTGACAGCATAACC	106 bp (1 CpG)
+215/+320 bp reverse	TAGTGGTTGGGTGGTAGATACA	

### Immunoblotting

For immunoblotting, rat tissues were rapidly homogenized in ice-cold radioimmunoprecipitation assay buffer (RIPA) Lysis and Extraction Buffer (#89900; Thermo Fisher Scientific) supplemented with 1 × Halt Protease and Phosphatase Inhibitor Cocktail (#78442; Thermo Fisher Scientific). The concentration of the extracted total protein was determined using the DC Protein Assay kit (Bio-Rad Laboratories, Inc) with bovine serum albumin as a reference standard. Equal amounts of proteins from all samples were subjected to reducing and denaturing gel electrophoresis on NuPAGE 4 to 12% Bis-Tris Mini Protein Gels (1.5 mm, #NP0335BOX, Invitrogen) or on Bolt Bis-Tris Plus Mini Protein Gels (8%, 1.0 mm; #NW00080BOX, Invitrogen). Subsequently, the proteins on the gel were transferred to a 0.2 μm polyvinylidene difluoride membrane using a Trans-Blot Turbo Transfer System (Bio-Rad) for 10 min at a constant current of 2.5 A and up to 25 V. The membrane was immediately submerged in 5% bovine serum albumin in Tris-buffered saline-0.05% Tween-20, pH 7.4 (TBST) and blocked for 1 h at 25 °C under constant agitation. The membrane was then incubated overnight at 4 °C with the following primary antibodies diluted in in TBST: rabbit anti-DNMT1 (1:500, #24206-1-AP, Proteintech Group, Inc), rabbit anti-DNMT3A (1:1000, #3598S, Cell Signaling Technology), rabbit anti-DNMT3B (1:1000, #72335, Cell Signaling Technology), rabbit anti-MeCP2 (1:1000, #C15310088, Diagenode), rabbit anti-TET1 (1:500, #GTX124207, GeneTex, Inc), rabbit anti-TET2 (1:500, #C15410255, Diagenode Inc), and mouse anti-TET3 (1:1000, #61395, Active Motif). The specificity of these primary antibodies has been previously validated (66, 67, 68, 69, 70, 71, 72). For the protein loading control, a rabbit anti-GAPDH antibody (1:1000, #5174S, Cell Signaling Technology) was used. Subsequently, a goat anti-rabbit horseradish peroxidase-linked antibody (1:5000, #7074S, Cell Signaling Technology) or anti-mouse horseradish peroxidase-linked antibody (1:5000, #7076S, Cell Signaling Technology) was applied to the immunoblots for 2 h at 22 °C. The protein bands on the membrane were visualized using the SuperSignal West Femto Maximum Sensitivity Substrate (#34094, Thermo Fisher Scientific) and the Odyssey Fc Imager (LI-COR Biosciences). The protein bands were then normalized to the corresponding GAPDH protein band on the same blot. ImageJ (https://imagej.net/ij/) software was used for the quantification of the protein bands.

### Chromatin immunoprecipitation-qPCR

ChIP was performed using the SimpleChIP Plus Sonication Chromatin IP Kit (#56383S, Cell Signaling Technology) following the similar methods described previously (62, 73). The PVN tissue was placed in ice-cold PBS containing protease inhibitor cocktail and cross-linked with 2% methanol-free formaldehyde for 15 min followed by quenching using glycine for 5 min on ice. After three PBS washes, the tissue was homogenized for 15 min in ChIP Sonication Cell Lysis Buffer supplemented with a protease inhibitor cocktail, and the cells were pelleted at 5000*g* for 5 min at 4 °C followed by nuclei isolation using ChIP Sonication Nuclear Lysis Buffer containing protease inhibitor cocktail. The chromatin fragmentation was performed in a Covaris M220 AFA-focused ultrasonicator (Covaris, LLC) at a 5% duty factor for 6 min. The efficacy of shearing (200–800 bp) was cross-checked by resolving on a 1.2% agarose gel at 30 V for 4 h. The lysate was clarified by centrifugation at 21,000*g* for 10 min at 4 °C and the supernatant with cross-linked fragmented chromatin was diluted at 1:4 ratio with ChIP buffer containing protease inhibitor cocktail. A 10% (volume) of the fragmented, diluted chromatin was stored at −20 °C for extracting input DNA. For each immunoprecipitation reaction, a 5 μg of diluted chromatin was incubated overnight at 4 °C with rotation by adding 5 μg of antibody against a target protein of interest. For the negative control, 1 μg of normal rabbit IgG (#2729S, Cell Signaling Technology) was used for immunoprecipitation. Following immunoprecipitation, 30 μl ChIP-grade protein G magnetic beads were added to each immunoprecipitation reaction tube and incubated for 2 h at 4 °C with rotation. Subsequently, the beads were washed twice with low salt buffer followed by another wash with high salt buffer and the bound chromatin was eluted using ChIP Elution Buffer for 1 to 2 h at 65 °C in a thermomixer. The eluted chromatin was de-crosslinked with 5 M NaCl and Proteinase K treatment for 2 to 8 h at 65 °C in a thermomixer followed by the purification of DNA using a QIAquick PCR Purification Kit (#28104, Qiagen). The purified DNA was subjected to real-time PCR using the promoter-specific primers (Table 4) mixed with the PowerUp SYBR Green Master Mix (Thermo Fisher Scientific) in a QuantStudio 7 Flex Real-Time PCR System (Applied Biosystems). The fast-cycling mode conditions (50 °C for 2 min, 95 °C for 2 min, 40 cycles of 95 °C for 1 s, and 60 °C for 30 s) were used to perform RT-PCR reactions. The rat negative control primer set 1 (#71024, Active Motif) was used as a negative control for each antibody immunoprecipitation reaction because these primers are specific for a gene desert region located on rat chromosome 3. The threshold cycle (CT) value in each group was normalized to the input using the following formula: (2^-ΔCT^) × 100%; where ΔCT represents (CT [ChIP] – (CT [Input] - Log_2_ (Input Dilution Factor).

**Table 4:** List of primers used in the ChIP-qPCR

Primer name	Sequence (5′→3′)	Amplicon size
*Agtr1a* promoter		
−9/+137 bp forward	CAGTTGGGAGGGACTGGATG	146 bp
−9/+137 bp reverse	GGACTCACCAGGGAATGTG	
*Slc12a2* promoter		
−72/+54 bp forward	CGCCGCGCCTTTAAACC	126 bp
−72/+54 bp reverse	GGCGCTCCCACTAAGGA	

### Statistical analysis

Data are presented as mean ± SD. The sample sizes used in the study were similar to studies we published previously (19, 22, 24, 58, 59, 62). The animals were assigned to the control and treatment groups in a 1:1 ratio as they became available; however, specific randomization methods were not used. Rats in which the microinjector tip was placed outside the PVN were excluded from data analysis. No assessment for outliers within the data was performed. Evaluation of data normality was conducted using the Shapiro-Wilk test prior to the selection of appropriate statistical tests. To determine differences between two groups, a two-tailed Student *t* test was utilized. For comparisons involving three or more groups, a two-way ANOVA was employed, followed by Bonferroni's *post hoc* test. *p* values less than 0.05 were considered to be statistically significant. All statistical analyses were conducted using Prism (https://www.graphpad.com/) software (version 9.3, GraphPad Software).

## Data availability

All the data supporting the findings in this research are available within the article.

## Conflict of interest

The authors declare that they have no conflicts of interest with the contents of this article.
